# A holistic approach for severe flexion contracture of bilateral hip, knee, and ankle joints in a neglected patient with prolonged knee–chest positioning on extreme undernourishment: a case report and review of the literature

**DOI:** 10.1186/s13256-022-03439-y

**Published:** 2022-05-24

**Authors:** Ahmad Jabir Rahyussalim, Muhammad Luqman Labib Zufar, Tri Kurniwati

**Affiliations:** 1 Department of Orthopaedic and Traumatology, Cipto Mangunkusumo National Central General Hospital and Faculty of Medicine, Universitas Indonesia, Jl. Pangeran Diponegoro No.71, RW.5, Kenari, Kec. Senen, Kota Jakarta Pusat, Daerah Khusus Ibukota, Jakarta, 10310 Indonesia; 2 Stem Cell and Tissue Engineering, IMERI Universitas, Jakarta, Indonesia

**Keywords:** Flexion contracture, Paraplegia, Traumatic spinal injury, Prolonged passive stretching, Case report

## Abstract

**Background:**

Flexion contracture in the lower extremity is a common finding in the patient with neuromusculoskeletal disorders. However, severe cases due to prolonged immobilization in knee–chest position are rarely established and remain underreported. This condition is associated with high morbidity and reduced quality of life, especially when it comes to neglected cases with missed injury and late presentation for adequate primary care and rehabilitative program. It remains a difficult challenge to treat, with no established treatment protocol. In addition, other factors related to psychological and socioeconomic conditions may interfere and aggravate the health state of such patients.

**Case presentation:**

A 19-year-old Javanese man presented with flexion contracture of bilateral hip, knee, and ankle joints due to prolonged immobilization in knee–chest position for almost 2 years following a traffic accident and falling in the bathroom. The condition had persisted for the last 3 years due to irrecoverable condition and lack of awareness. In addition, the patient also presented with paraplegia at level L2–S1, dermatitis neglecta, multiple pressure ulcers, community-acquired pneumonia, and severe malnutrition. Prolonged and sustained passive stretching with serial plastering were performed in the patient. By the time of discharge, patient was able to move and ambulate using wheelchair. Progressive improvement of range of motion and good sitting balance were observed by 3-month follow-up.

**Conclusion:**

A combination of surgery and rehabilitative care is required in the setting of severe flexion contracture. Passive prolonged stretching showed a better outcome and efficacy in the management of flexion contracture, whether the patient undergoes surgery or not. However, evaluation of residual muscle strength, changes in bone density and characteristic, and the patient’s general and comorbid conditions must always be considered when determining the best treatment of choice for each patient to achieve good outcome and result. A holistic approach with comprehensive assessment is important when treating such patients.

## Introduction

Joint contracture is a common clinical finding related to any kind of neuromuscular disease or disorder. It may occur following traumatic accident, inflammatory conditions, spinal cord or brain injury, and specific neuromuscular diseases (Duchenne and Becker muscular dystrophies, Emery–Dreifuss muscular dystrophy, congenital muscular dystrophies, congenital myopathies, arthrogryposis, spinal muscular atrophy, etc), but prolonged immobilization remains the most common cause [1–5]. Furthermore, lower limb contractures, especially flexion contracture, are reported to be more prevalent and commonly found compared with upper limb contractures. Such patients present with minimal to excessive deformities, hampered motoric function, prolonged and chronic pain, sleep disturbance, and breakdown of the skin, ligaments, and tissue structures. This condition may also be associated with muscle wasting and progressive bone osteoporosis, and lead to debilitating issues, as well. It eventually leads to a higher morbidity rate and reduced quality of life in such patients [6–8].

Moreover, other factors such as the psychological and socioeconomic conditions of the patient may interfere and aggravate the health state and disease progression. Accessible healthcare, advances in emergency and trauma care, and psychological support become important factors [9–11]. However, missed injuries, late presentation, inadequate primary care, premature discharge without adequate rehabilitation care, poor socioeconomic condition, and low educational level remain as reasons for why patients are not well diagnosed or treated. Missed diagnosis and delays in prompt treatment will give rise to delayed injuries with poor outcomes and prognosis [12–15]. We report herein our experience in treating a rare case of severe flexion contracture involving bilateral hip, knee, and ankle joints in which the patient was lying supine and neglected in prolonged knee–chest position. According to the authors’ knowledge, this is the first such case to be reported, never mentioned before in other studies. The causes, complications, and treatment challenges in managing the patient are discussed comprehensively.

## Case presentation

A 19-year-old Javanese man was admitted to our spine unit in the Orthopedic and Traumatology Department of Cipto Mangunkusumo National General Hospital with severe flexion contracture of bilateral hip, knee, and ankle joints due to prolonged immobilization in knee–chest position for almost 2 years. The patient had a history of falling from a motorcycle in a road traffic accident 3 years ago, followed by a decrease in functional movement of both legs with leg pain. There was no finding of radiculopathy, reduction of neck range of movement, or sensory deficit. Six months following this first traumatic incident, the patient fell in the bathroom with supine position. The patient was still able to move his legs. However, he was not able to stand or walk. The patient mobilized by dragging his body using both hands.

His family as care-givers took the patient to a traditional massage therapist as the initial management, without seeking any adequate medical treatment at a health center. The patient was put in knee–chest position to decrease the pain, and it turned out that this position had made the patient feel more comfortable. The medicine man and the those around the patient considered placing him in reduced movement and stretching stimulation, believing that this method would allow his injuries to heal. The patient was bedridden at best, without any other kind of mobilization and limited daily activities. This condition gradually worsened over the last 1.5 years. A major decrease in body weight accompanied by multiple painful and itchy skin lesions were found to be due to insufficient nutritional support and poor hygiene. The patient was then referred to the regional general hospital and diagnosed with scabies, but no bony and spinal cord abnormalities were found. Faced with a lack of adequate medical treatment and progressive deterioration of his condition with worsening of the flexion contracture and skin condition, as well as a decrease in body weight, the patient was eventually taken by a nongovernmental organization to our spine unit. The patient had no remarkable past illness, medication, family, or psychosocial history.

**Fig. 1:**
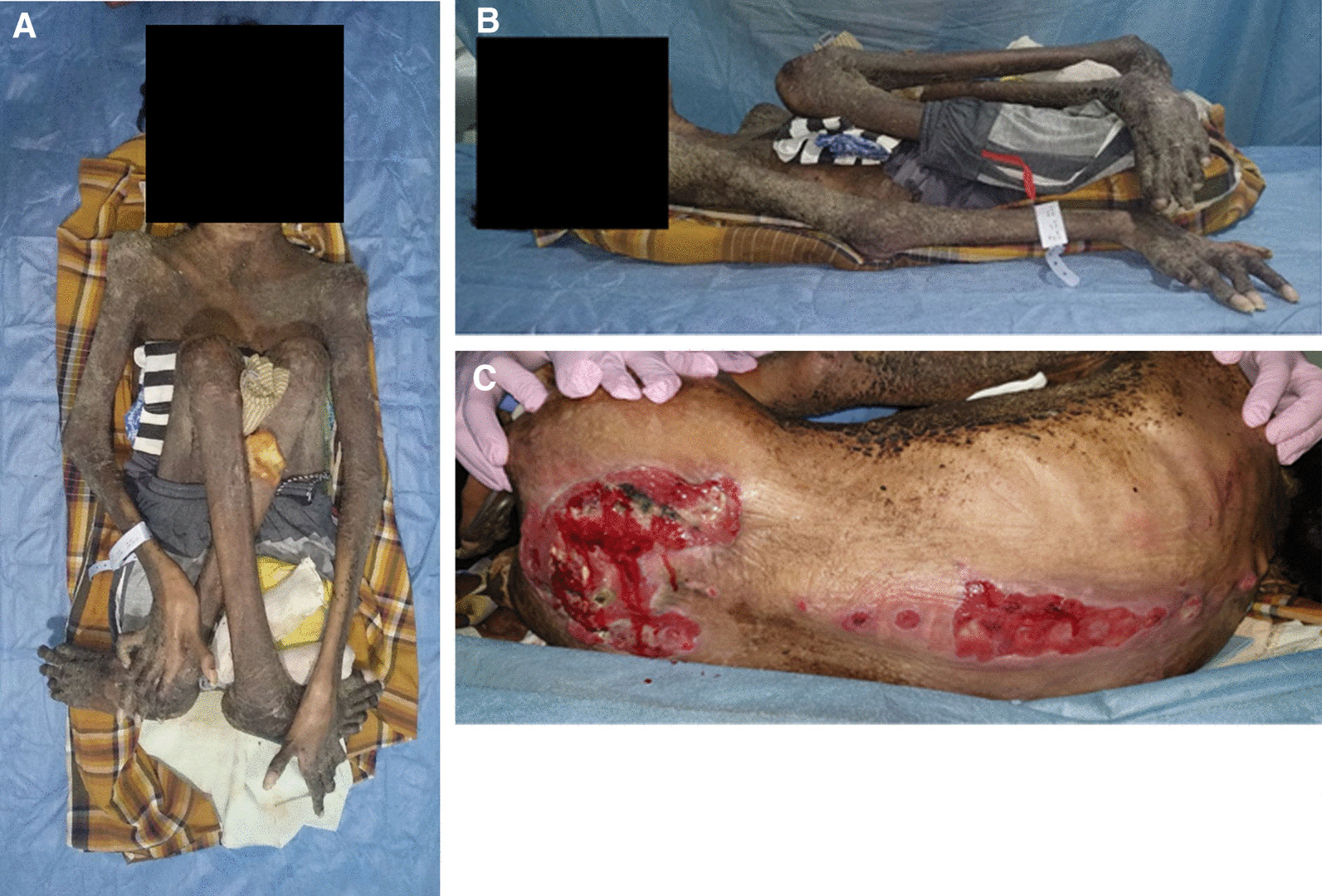
Patient presented with flexion contractures of bilateral hip, knee, and ankle joints (**A** and **B**) with associated condition of multiple pressure ulcers at sacral, lumbar, and bilateral femoral shaft region (**C**)

Our physical examination showed no anemic conjunctiva, icteric sclerae, and bruises over thoracic region. There was symmetric movement of both sides of the lungs during inspiration and expiration, with vesicular breath sounds and normal heart sounds plus no rales, murmur, or gallop. Abdominal examination was difficult to perform due to his poor nutritional status, skin condition, and flexion contractures in the lower extremities. The local state of the back showed no deformity. However, there were open wounds of multiple grade 3 pressure ulcers on the thoracic, lumbar, and sacral region (Fig. 1). Tenderness on palpation was found with pain severity at level 2/3 on a visual analog scale. The patient presented sensory deficit at levels C5–T1 and L2–S1 with marked hypoesthesia below T1 level and associated paraplegic motor deficit over C5–T1 and L2–S1 level on neurological examination.

The preserved overall magnitude of muscle strength in the patient was 2/2 at C5–T1 level and 0/0 at L2–S1. Range of movement for hip, knee extension, and ankle extension, eversion, dorsiflexion, and plantarflexion were limited due to contracture. Reflexes over patellar tendon and Achilles tendon were difficult to evaluate due to the contractures in the lower extremities. Urinary incontinence without fecal incontinence and no pathological reflexes of Babinsky group nor clonus were discovered on examination. We further investigated the patient by using laboratory and radiography studies. Blood albumin and hemoglobin level were at low level (9.0 g/dL and 1.9 g/dL, respectively). Besides, leukocyte and thrombocyte counts were increased (11,110/µL and 977,000/µL, respectively). Culture of skin swab on the base of decubitus ulcers showed *Klebsiella pneumoniae* and *Escherichia coli* as the main bacteria in the wounds. Plain radiography of thorax, pelvis, lumbar vertebrae, bilateral cruris, and bilateral femur showed no fracture or destruction, except for flexion deformity of bilateral genu and ankle, with marked bilateral ankle inversion.

**Fig. 2:**
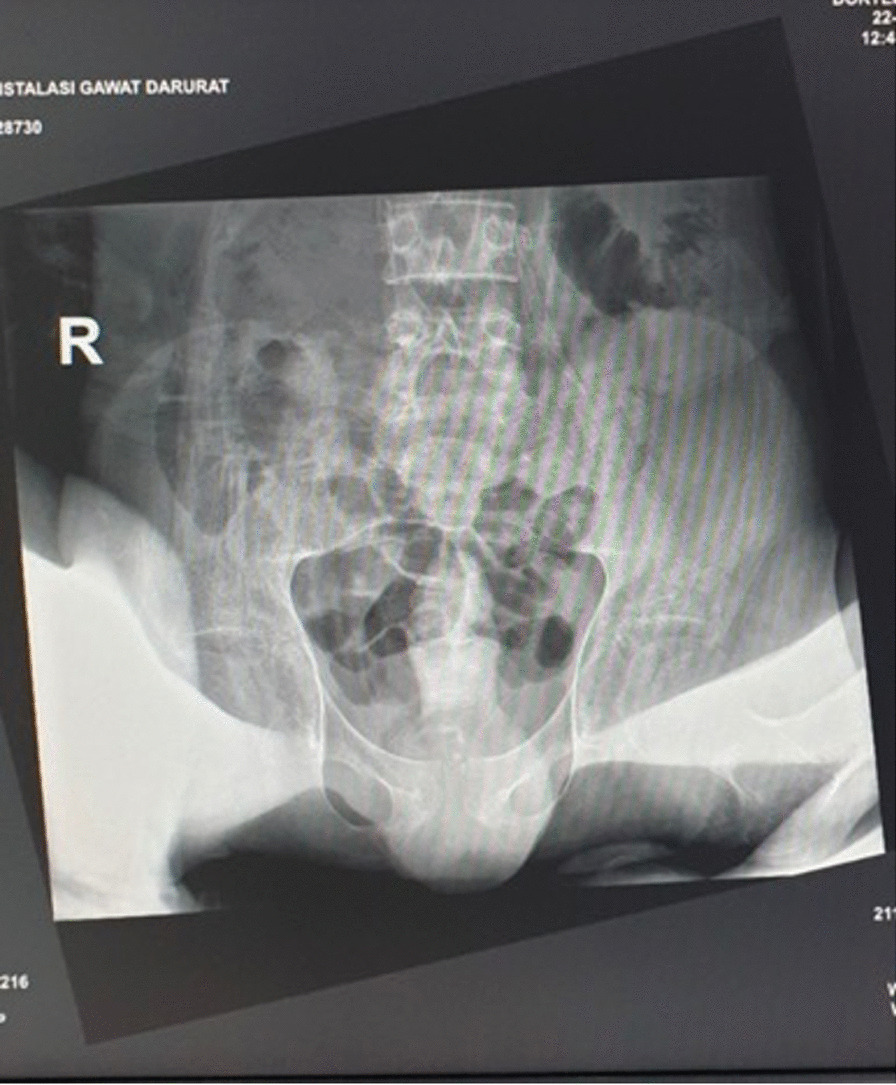
AP plain radiographs of pelvis, showing decreased density and porotic bone without fracture or bone destruction

**Fig. 3:**
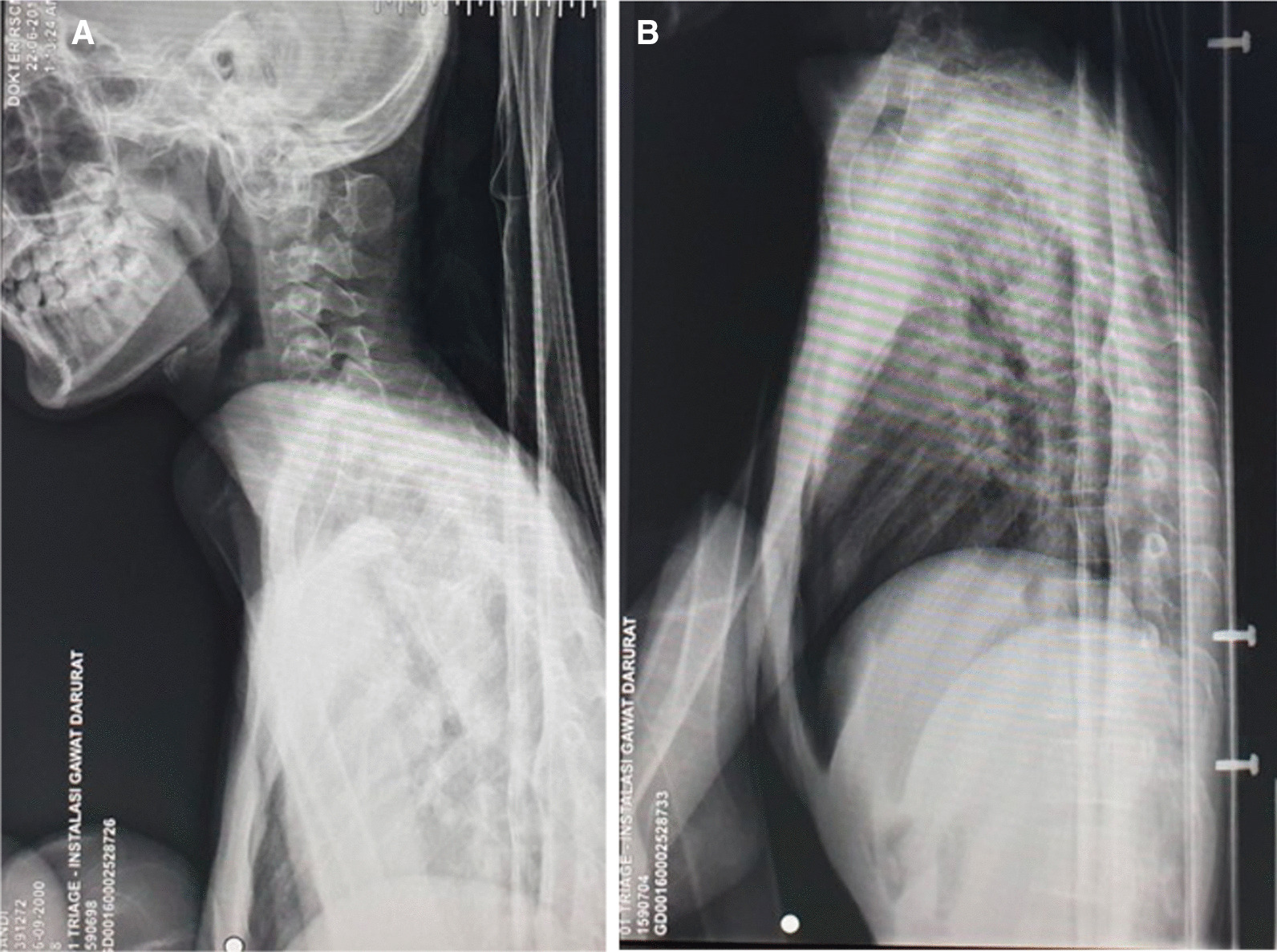
Lateral plain radiographs of cervical spine (**A**) and thorax (**B**), showing decreased density and porotic bone without fracture or bone destruction

**Fig. 4:**
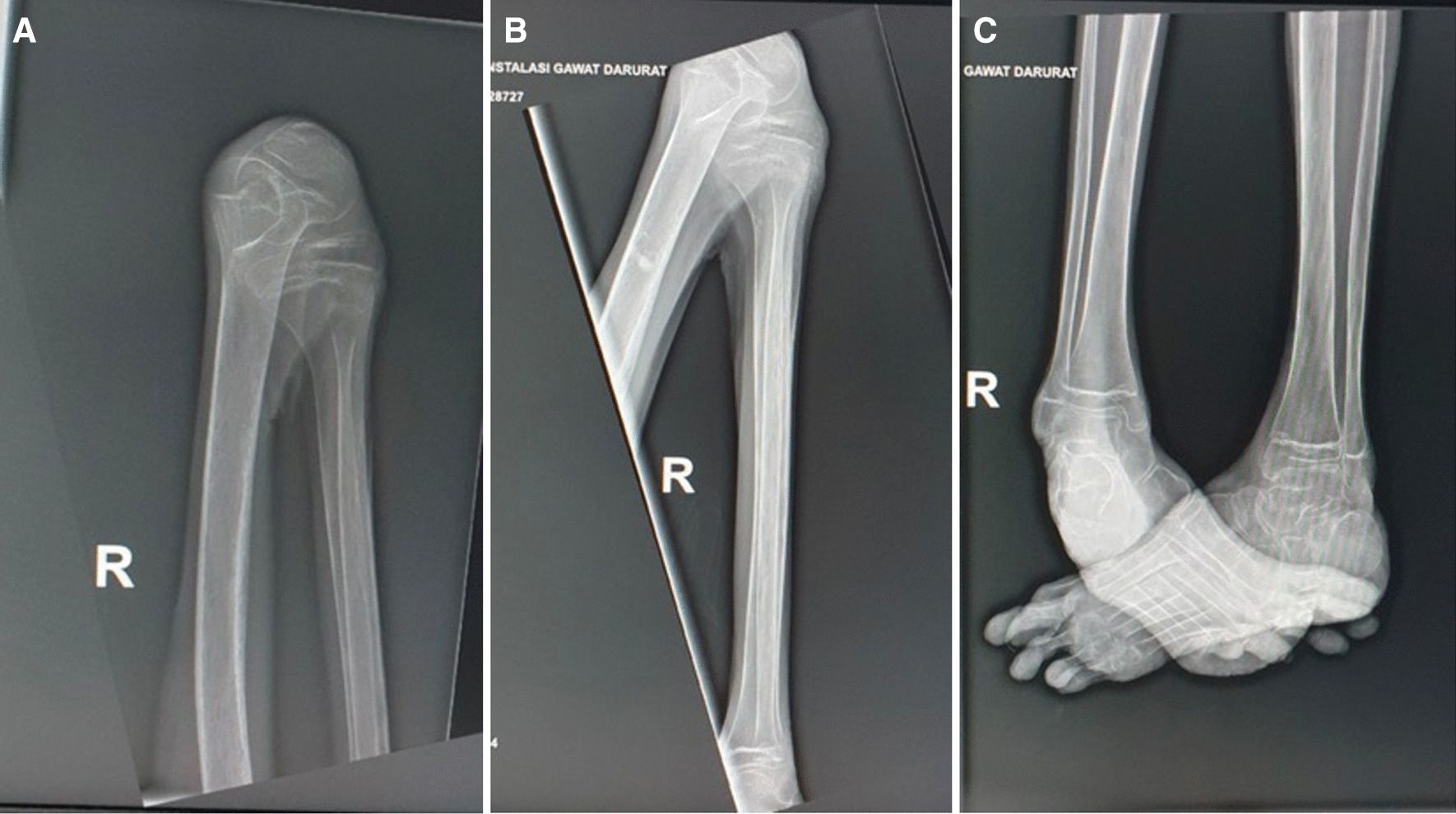
Lateral plain radiographs of femur (**A**, **B**) and AP plain radiographs of cruris bilateral (**C**), showing decreased density and porotic bone, flexion deformity of genu and ankle, and bilateral ankle inversion without fracture or bone destruction

**Fig. 5:**
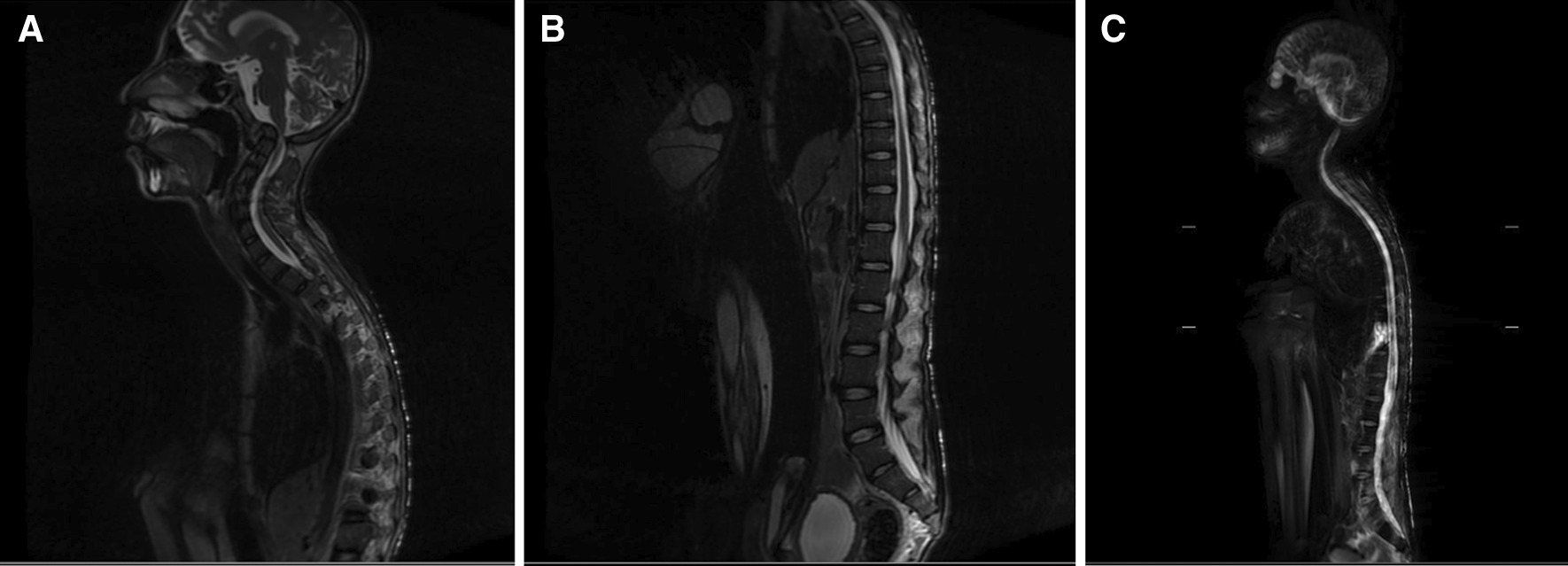
Sagittal MRI of cervical (**A**), thoracic (**B**), and whole spine (**C**) showing no remarkable cord compression or myelomalacia changes, with cervical hyperextension and thoracic kyphotic and scoliotic deformity

All plain radiographs showed porotic and decreased density of the bone (Figs. 2, 3, 4). Whole-spine magnetic resonance imaging (MRI) with contrast (Fig. 5) showed hyperextension deformity over cervical vertebral column, kyphotic and scoliotic deformity over thoracal vertebral column, without any increase in contrast intensity related to pathological condition across corpus vertebral and intra medulla. MRI study did not showed any significant compression over the cord or myelomalacia changes. We diagnosed this patient with severe flexion contracture of bilateral hip, knee, and ankle joints due to prolonged immobilization in knee–chest position for almost 2 years with associated medical conditions of dermatitis neglecta, multiple pressure ulcers grader 3 in sacral region, grade 2 at bilateral femoral shaft and lumbar region, and dyspnea due to community-acquired pneumonia. Besides, this patient also presented with severe malnutrition.

**Fig. 6:**
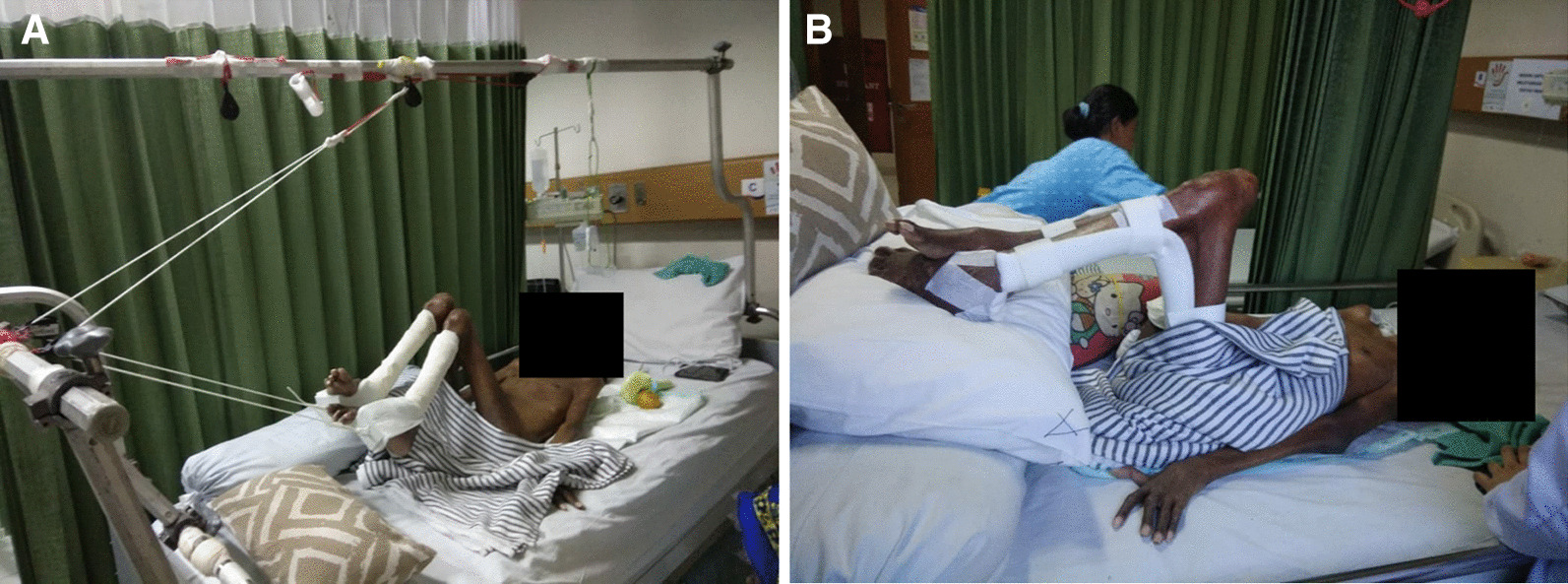
Applied prolonged and sustained passive stretching (**A**) followed by serial plastering and bracing (**B**) in the patient

We were highly concerned about and gave attention to this case because of its complexity, requiring comprehensive multidisciplinary management, not only from the orthopedic and traumotology field, but also from other departments in our center, Cipto Mangunkusumo National General Hospital. We involved our internist and clinical nutritionist colleagues for managing the patient’s general condition, especially related to the comorbid condition of pulmonary infection and severe malnutrition. Regarding skin hygiene and wound care in treating the skin lesions and multiple pressure ulcers, we involved our dermatologist and plastic surgeon colleagues. In our orthopedic surgical field, with the help of rehabilitative colleagues, we performed passive stretching and movement 3–5 times a day with a total time of 30–60 minutes daily for all joints in the upper and lower extremities. Besides, we also performed sustained stretching using continuous traction over lower extremity, consisting of hip, knee, and ankle joints on both sides of the legs (Fig. 6). The goal of the treatment in this patient was to gradually re-enable independent daily activities. By the time of discharge in this first period of treatment, the patient was able to move and ambulate using a wheelchair with improved contractures in both extremities. The patient was reported to have good adherence and tolerability regarding rehabilitative care planning exercises in the home given by the time of discharge. Marked improvement of hip and knee ROM was observed. Maintaining good sitting balance was established by 3-month follow-up. There was no worsening of contracture or general state of the patient. The patient is planned to have release surgery for hip, knee, and ankle contractures following improvement in his general and comorbid conditions of community-acquired pneumonia, severe malnutrition, skin lesions, and pressure ulcers.

## Discussion

Joint contracture, especially flexion contracture in the lower limb, is one of the most common complications in the musculoskeletal system, aside from muscle weakness and atrophy, soft tissue changes, disuse osteoporosis, and degenerative joint disease following prolonged immobilization. It is commonly found when a patient is put in static positioning for a continuous length of time, such as in patients with wheelchair reliance or bedridden patients with chronic illness or acute critical illness. Besides, immobilization used as critical treatment in fractures, joint dislocations, and ligament injuries should also be considered [1–4]. This condition is consistent with our patient in whom immobilization is thought to be the main cause of contracture. Our patient was put in prolonged knee–chest positioning for almost 2.5 years, resulting in severe flexion contracture of bilateral hip, knee, and ankle joints. Pathological change is reported to begin after 2 weeks of immobilization, while more prolonged time is associated with more extensive contractures [16, 17]. Flexion contracture in the lower limb contributes significantly to disability in activities of daily living due to mobility limitation, decreased functional motor function, and reduced range of motion. Moreover, severe and chronic cases of this condition are associated with debilitating issues in which disease progression may affect not only the neuromusculoskeletal system but also other organ systems, especially respiratory, cardiovascular, and genitourinary [18–20].

The pathophysiology of joint contracture is multifactorial and immensely diverse. It involves both histopathological changes of the various neuromusculoskeletal structures and environmental factors. Negative alteration of healthy connective tissue is related to prolonged immobilization. Homeostasis of mechanical properties of the connective tissue and its function is disturbed with the loss of mechanical stimulation of the joint. Regarding the type of anatomical structures and tissues involved in the restriction of range of movement, muscles, tendons, fascia, capsule, ligaments, bone, cartilage, and skin may play significant roles. These tissues can be classified into two main structural components leading to joint contracture in immobilization, viz. myogenic and arthrogenic contracture components. Myogenic contracture plays an important role and becomes the principal component in the early stage. It involves mainly muscle, tendon, and fascia changes. Arthrogenic contracture, on the other hand, accounts for the later stage, in which changes are found across cartilage, joint capsule, bone, and ligament structures [21–23].

When a joint goes through continuous and prolonged immobilization, skeletal muscle will undergo disuse atrophy. Reduced cross-sectional area (CSA) and shortened muscle fiber were observed under the microscope. Besides, a decrease in muscle protein synthesis with enhancement of proteolysis has also been reported [24, 25]. This condition is associated with the catabolic phase, involving five main proteolytic pathways, including the ubiquitin–proteasome-dependent pathway, caspase system pathway, matrix metalloproteinase pathway, autophagy–lysosomal pathway, and Ca^2+^-dependent pathway. With an immobilized muscle put in a shorter position, loss of sarcomere will be present as an adaptation to muscle functional length [24, 26]. In addition, fibrosis over the skeletal muscle tissue is also found as pathological changes affecting further progression of muscle contracture. There will be accumulation of fibrotic tissue in the perimysium, epimysium, and endomysium because of overexpression and overproduction of collagen, with type 1 primarily followed by type III collagen. Fibril arrangement will adapt to be more circumferential with shortening of muscle fibers. Changes with increased intramolecular and intermolecular cross-linking also restrain skeletal muscle extensibility. Besides, interleukin (IL)-1β and transforming growth factor (TGF)-β1 upregulation by macrophages with significantly higher level of protein and messenger RNA (mRNA) level of hypoxia inducible factor-1α (HIF-1α) expression is associated with fibroblast differentiation in immobilization-induced skeletal muscle fibrosis [24, 27, 28].

Previous studies have reported that ubiquitin–proteasome-dependent proteolysis and the caspase system pathway play a key part in the development of contracture regarding muscle atrophy in disuse skeletal muscle. This is indicated by overexpression and an increase in the amount of muscle atrophy F-box protein (MAFbx or atrogin-1) and muscle ring finger-1 protein (MuRF-1) as muscle-specific ubiquitin-protein ligase in muscle atrophy due to disuse of skeletal muscle in immobilization [29]. Besides, major signaling activators of MAFbx and MuRF-1, protein kinase B (Akt), insulin-like growth factor 1 (IGF-1), and phosphatidylinositol 3-kinase (PI3K) are increased prior to upregulation of MAFbx, MuRF-1, and other proapoptotic genes [30–32]. Inhibitor of nuclear factor kappa-B kinase (IKKβ/NF-κB) may also be an important pathway, specifically for MuRF-1 [33]. The use of MG132 as an inhibitor of the NF-κB pathway demonstrated decreased expression of MuRF-1 [34]. Eukaryotic translation initiation factor 3-f (eIF3-f) and myogenic differentiation (MyoD) become two main muscle regulatory factors targeted by muscle-specific E3s. EIF3-f and MyoD play an important role in activation of the proliferation and differentiation of myogenic satellite cells into myoblasts, which further fuse to form new muscle fibers [35–37]. In addition, the role of the ubiquitin–proteasome-dependent system in degeneration of myofibrillar protein works synergistically with the calpain system [38].

Regarding the caspase system, an increase in caspase-3 level is reported during immobilization. Caspase itself is a cysteine-rich enzyme that targets aspartic acid residue in specific protein. Caspase-3 showed a significant role in the initiation of muscle disuse atrophy through apoptosis and inflammatory processes [39, 40]. In disuse of skeletal muscle due to immobilization-induced joint contracture, tissue inhibitor of metalloproteinase (TIMP)-1 and -2, and other MMP pathway are associated with atrophy in remobilization. Their levels were increased by 30- and 2-fold, respectively, in immobilized anterior tibialis muscle at day 8 of observation and remained raised until day 5 of recovery. This is followed by increased MMP-2 and MMP-14 mRNA levels at days 1–9 of recovery [25, 41]. Regarding the autophagy–lysosomal pathway, lysosomal cathepsin will break down membrane proteins, together with receptors, ligands, and transporters. The level of the LC3-phosphatidylethanolamine conjugate (LC3-II)/cytosolic form of microtubule-associated protein light chain 3 (LC3-I) was observed to be higher in immobilized muscle. An increase in the value of this ratio was associated with increased autophagy–lysosomal activity, since LC3-II was mainly found in autophagosome membranes [42].

Within a later stage of prolonged immobilization, changes over arthrogenic structures may also account for further contracture progression. However, early changes are associated with proliferation of fibro-fatty tissue invading joint space. In a diarthrodial joint, the capsule plays a key role in limiting the range of motion. It is made of a densely fibrous structure, mainly composed of collagen protein. Fibrotic changes of the joint capsule with an increased level of type I collagen have been reported in immobilized limbs. Besides, a decreased level of glycosaminoglycan following collagen fiber disorganization will further result in a decreased water level and accumulation of collagen crosslinking. Other reported changes include decreased proliferation of synoviocytes with disruption over normal length of synovial intima [43, 44]. Genomic study and analysis have also reported upregulation of chondroadherin, angiogenic inducer CYR61, and SRY-box with decreased casein mRNA. These changes were found in tissue fibrosis over posterior capsule of patients with OA and contractures [43].

Other proposed mechanisms in regard to the induction of contracture in our patient are due to muscle weakness and spasticity following spinal trauma. We consider these mechanisms since our patient had a history of falling from a motorcycle in a road traffic accident 3 years ago and falling down in the bathroom in supine position 2.5 years ago, leading to a suspected spinal cord injury despite normal findings reported in radiological studies. This condition may be referred to the spinal cord injury without radiographic abnormality (SCIWORA) or spinal cord injury without radiographic evidence of trauma (SCIWORET) concept in which a patient with normal findings on plain radiographs and computed tomography (CT) scans may present with neuromuscular symptoms (sensory or motor deficit, or both). The difficulties in establishing the diagnosis are associated with its rarity and broad variety of symptoms. This condition presents commonly following a traumatic accident or degenerative process. Neuroimaging studies, especially using magnetic resonance imaging, allowed better visualization of damage and structural abnormality of spinal soft-tissue and intramedullary lesions [45, 46]. Nevertheless, microscopical changes with chronic disease progression in our patient also need to be considered and take into account, since confirmation using MRI studies reported normal findings.

The main mechanisms of primary injury result from impact, transient or complete compression, distraction, and laceration or transection, with impact together with preserved compression being the main form of injury [47, 48]. Further changes of secondary injury will follow during disease progression. This phase consists of vascular damage, ionic imbalance, neurotransmitter accumulation (excitotoxicity), free radical formation, calcium influx, lipid peroxidation, inflammation, edema, and necrotic cell death in the acute period. Apoptosis, demyelination of surviving axons, Wallerian degeneration, axonal die-back, matrix remodeling, and evolution of a glial scar occur thereafter in the subacute period. Furthermore, formation of a cystic cavity, progressive axonal die-back, and maturation of the glial scar will present in the chronic period throughout the injury site. Severity and location, and whether the lesion lies over the spinal cord completely or not, influence the clinical outcome of the patient [47].

Neurological impairments, presented as paraplegia, hemiplegia, tetraplegia, or muscle paralysis, are some of the most debilitating issues related to traumatic spinal injury. This condition may progress and have an impact on the emergence of other complications, especially related to the musculoskeletal system. Contracture of the extremities in traumatic paraplegic patients is a common finding. With muscle weakness due to spinal injury, there may be a disruption of balance and stability between agonist and antagonist muscles [47, 49, 50]. Lower-limb flexion contracture and equinovarus deformity are more susceptible to develop as the patient becomes weaker during progression of the disease and spends more time in the sitting position. Besides, denervation will further result in loss of contraction stimulation with size reduction and necrosis of muscle fibers. Muscle spasticity may also be associated with contracture progression development in the traumatic patient, whether it affects the brain or spinal cord structure, resulting in upper motor neuron lesions. Muscle stiffness and imbalance with deformed posture, increased binding between actin and myosin, increased connective tissue with decreased elasticity, and shortening of the myofibril due to sarcomere loss will follow the spastic contraction in the patient.

Psychological and social conditions also play a key role and become significant factors regarding disease progression in our patient. Health beliefs and expectations of the patient are associated with vulnerability to illness. Besides, the patient will adapt and behave to reduce the risk of illness in regard to his/her health beliefs and expectations, which develop throughout their life. Culturally determined health beliefs, which are still commonly found in society, especially in our case, in addition to formed individual perception, may influence coping behaviors regarding musculoskeletal problems [51]. Our patient comes from a family and society with low educational level. He had already opted for and preferred another form of traditional massage treatment before presenting to the healthcare facility. Moreover, the traditional medicine man also emphasized fear-avoidance beliefs, in which physical activity and work are associated with disability and pain deterioration [52].

This further influenced the beliefs of the patient, his family, and environment regarding his care. This concept can also be associated with kinesiophobia, defined as “excessive, irrational, and debilitating fear of physical movement and activity resulting from a feeling of vulnerability to painful injury or re-injury” [53]. When a patient is placed under prolonged rest and immobilization, this state of freedom from physical and mechanical stressors is believed to allow injuries to heal. However, as we know, movement and stretching stimulation are important to improve range of motion and prevent contracture formation. The presence of fear-avoidance beliefs becomes an indicator of poor prognosis and must be well managed. In the long term, this condition will trap the patient in a vicious cycle of increased fear of more pain and disability, which further causes worse disuse, disability, and depression [51, 54].

The social aspect, including social support, also has a significant impact on physical health in regard to recovery from musculoskeletal injury. More intimate and trusting relationships are associated with more beneficial effects of support, as suggested by the Convoy model. Better physical health and recovery were reported in individuals with strong support from family and friends. A closer, more intimate, and stable relationship is more convenient and beneficial for support. A lack of social support, whether that be no social circle, difficulty in accessing a support network, or a strong personal belief in independence that does not require social support, resulted in a higher complication rate and a hampered recovery process [55, 56]. Functional support from family members is mainly related to material and instrumental aid, while emotional support is more frequently provided by friends. However, we also need to emphasize patient personality traits influencing his/her beliefs regarding the need for and and perception of social support.

In our case, the patient’s condition was reported to have worsened following the treatment obtained from traditional massage therapy, with no improvement for almost 2.5 years. The patient and his family were put in jeopardy by pessimistic beliefs and low expectations about disease prognosis. The patient’s mother was the only family member to live with and care for him, yet she started to neglect him with the worsening of his condition at the end. No support was provided by the patient’s father or any other family members. Our patient has low socioeconomic status and lacks financial and psychosocial support. Furthermore, he lives in a rural area with difficult access to healthcare facilities, adequate infrastructure, and trained manpower. After 3 years of neglect of his condition, some of his neighbors who cared for and worried about the patient’s condition and prognosis finally asked a humanitarian and social aid institution for help to bring and refer him to our center at Cipto Mangunkusumo General Hospital to obtain the required treatment.

In severe cases of hip flexion contracture exceeding 90°, the patient and physician are faced with a difficult challenge with no fixed treatment protocol and other associated chronic complications, including pressure sores, hyperlordosis, or even scoliosis. Thorough and conscientious physiotherapy combined with surgery play an important role in the management of this condition [6]. Nicodemo *et al.* reported five cases of hip flexion contracture treated with either Girdlestone or replacement arthroplasty, or myoarthrolysis [57]. Besides, there were 12 proximal femoral resection procedures in children with spastic and painful hip dislocation and paraplegic flexion contracture [58]. Another proximal femoral resection procedure was reported by Bhattacharyya *et al.* regarding longstanding hip flexion contracture with flaccid paraplegia in a 20-year-old female student. Immediate straight hip joint with the ability to sit, lie on her back, and mobilization using a wheelchair was reported [6]. Myotomy across iliopsoas and obliquus externus showed good results. Hip replacement arthroplasty is another common procedure with satisfactory outcome. Complete extension of hip and knee with some of the patients returning to walking were obtained. However, high rates of recurrent dislocation of the hip, infection, implant loosening, blood loss, and osteoporosis of the bone are associated with hip replacement [59, 60].

Regarding knee flexion contracture with severe presentation, a combination of knee arthroplasty followed by serial casting and rehabilitative knee mobilization plays an important role in achieving a favorable outcome. However, residual flexion contracture of 7–10° was found, especially in severe cases with contracture greater than 80–90° [61, 62]. Additional distal femur resection and Z-plasty procedure over iliotibial tract and biceps tendon after knee arthroplasty may be needed in severe cases. Performing a Z-plasty procedure before knee arthroplasty is associated with a high rate of tibial dislocation [63]. Postsurgery serial casting or other orthotic management by gradual and controlled knee extension with regular continuous passive stretching resulted in increased range of movement. Besides, casting is preferred to be applied continuously for no longer than 5 weeks due to the increased stiffness and thermal injury complications [64]. Regarding nonoperative treatment, serial casting and wedging can extend the knee to a full range of extension [65, 66]. Moreover, contractures over the ankle region, whether plantar or dorsiflexion, with or without inversion or eversion of the soles, are commonly found in patients with central nervous system injury. The prevalence was reported to be as high as 57% in patients with spinal injuries [67]. Combined treatment to achieve terminal stretch may involve several methods of passive stretching, active ROM, antagonist strengthening, and release surgery. Compared with short duration with high tension strength, passive prolonged stretching is reported to have better outcome and efficacy. Adjustable dynamic orthoses provide good and effective results in the treatment of contractures. Besides, sustained stretch can also be achieved by using serial casting [68].

However, there are several factors one must consider before deciding on and performing surgery as the main management of contracture in such patients. First, residual muscle strength should be taken into account. With appropriate and adequate rehabilitative training, nerve and muscle can regain their functional capacity and strength through the preserved plasticity mechanism. We also need to evaluate whether there are marked changes of the density and characteristics of the bone. Loss of weight bearing and contraction with associated increased catabolic state result in osteoporosis. The rate of bone loss was reported to be 5–20 times higher compared with metabolic diseases. Rigorously intensive postoperative rehabilitation combined with muscle imbalance is associated with an increased risk of fracture. Higher rates of periprosthetic fractures and implant loosening were also reported. Furthermore, the patient’s general condition and associated comorbid disease must always be taken into account. Paraplegic patients have impaired microvasculature over skin and subcutaneous tissues, with long-lasting proprioception and exteroception damage. Occlusion across capillaries and small vessels followed by metabolic alteration may cause improper wound healing and further deterioration of the postoperative general state of such patients [69].

**Fig. 7:**
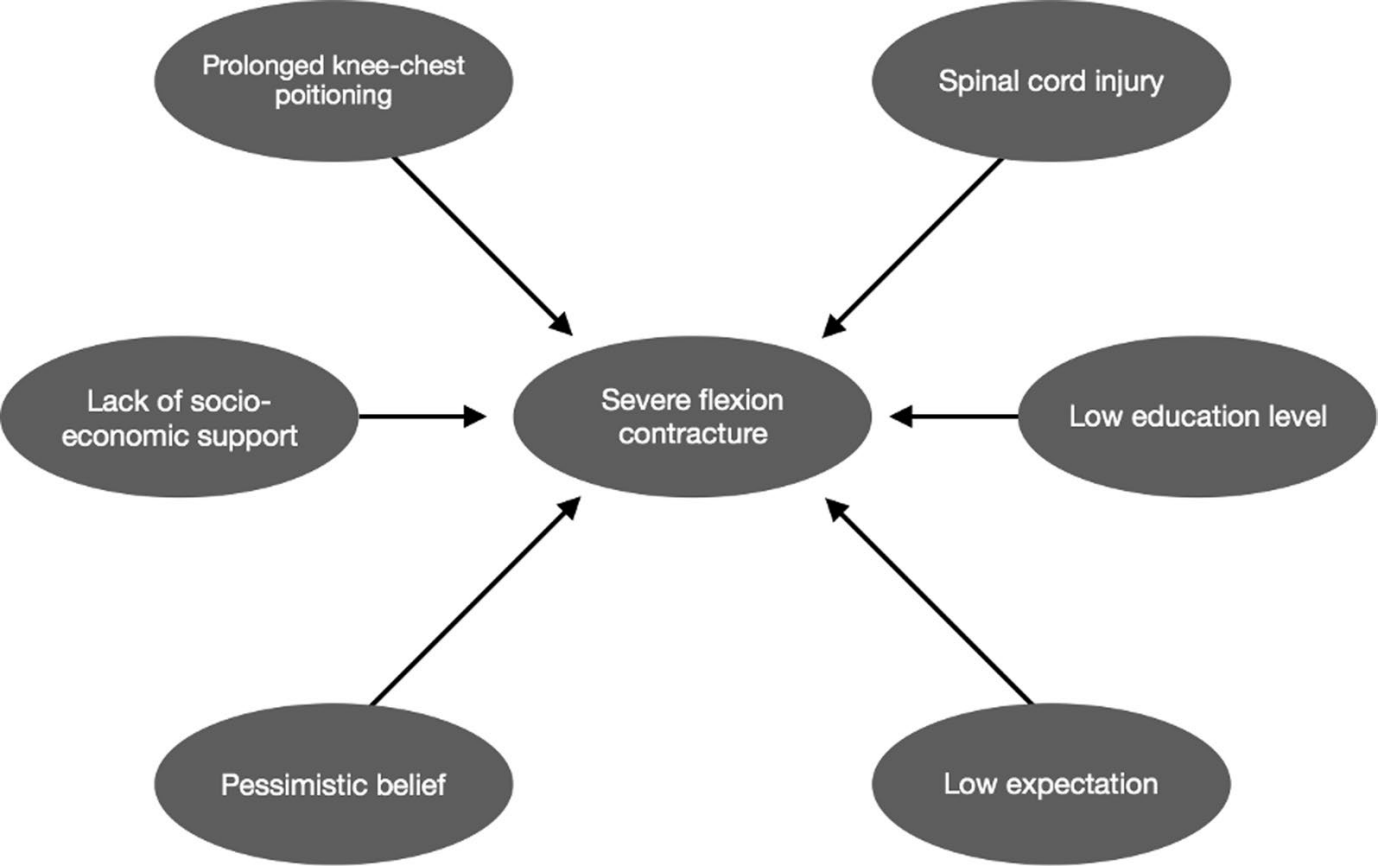
Biopschyosocial model affecting disease progression in our patient

Regarding our case, the patient presented with missed injury and overlooked diagnosis, mainly due to the late presentation to healthcare facilities with inadequate treatment in the initial contact. Furthermore, the lack of adequate further follow-up, sociopsychological support, and rehabilitative care, subsequent to the discharge from the initial treatment, also played an important role in the deteriorating progression toward the worsened condition of the patient (Fig. 7). Rehabilitation with physical therapy became the preferred treatment modality for the patient. We implemented several principles of physical therapy, including passive stretching of muscles and joints, promotion of extension positioning of the limb, splinting or serial plastering/casting to manage, prevent, and delay further progression of the contracture in our patient.

We performed passive stretching with assisted continuous skin traction and serial plastering. Passive stretching 3–6 times a day with a total duration of 30–60 minutes was conducted at the beginning of the first 2 weeks, followed by continuous traction for 3 weeks. Serial plastering and bracing were changed toward the new limit of range at regular times. In the end of the fifth week, range of movement (ROM) in our patient had improved with a final ROM of hip flexion of more than 90° bilateral, 110° in right knee, and 115° in left knee. At 3 months postdischarge, hip flexion ROM was 90° bilaterally, with knee extension ROM increased to 25° bilaterally. The patient was not burdened by a bedridden condition anymore and could ambulate using a wheelchair.

Although the effectiveness and success of passive stretching are influenced by the characteristics of the contracture and predisposing factors in each patient, prolonged and sustained passive stretching over the contracture region had showed favorable outcomes compared with shortened duration. Daily stretching of 30 minutes over the immobilized joint was reported to prevent sarcomere loss. Another study showed that sustained stretching of 50 minutes was effective to counteract the dynamic changes in muscle and connective tissue [49]. Prolonged stretching with the help of other modalities may be considered when a passive movement pattern has failed to achieve the expected ROM of the contracture. Besides, the presented static postures sustained by spasticity of the neuromuscular changes must be taken into account as well [49, 68].

Application of serial plastering plays an important role in maintaining continuous immobilization. In addition, antagonist muscle of the stretched muscle may suffer changes due to the shortening and the emergence of minimal atrophy. The use of serial plastering in a lower limb flexion contracture has showed positive outcome with full extension accomplished in the best case [64, 70]. The length–tension curve may shift toward the flexion or opposite direction of the contracture as well. Cusick *et al.* reported the application of serial casting in a case study, showing regained full extension of the right leg with residual 5° contracture of the left leg prior to bilateral 40° flexion contracture. Increased ankle range of dorsiflexion was reported by Brouwer *et al.* in the 3 weeks after serial casting. Plantar-flexor strength was preserved in the study [49]. Increased range of dorsiflexion and decreased tone of plantar-flexor were also observed in a patient with cortical lesion undergoing serial casting [71]. However, recurrence may occur due to persisting spasticity. Besides, prolonged immobilization in serial plastering may cause alter the muscle/tendon ratio with a length–peak torque shift of the muscle, further resulting in muscle atrophy, muscle weakness, and decreased elasticity of the connective tissue.

Other major complications associated with the neglected case of prolonged knee–chest positioning in our patient were severe malnutrition, CAP, multiple skin lesion with pressure ulcer grade 2 and 3, and poor skin hygiene. Besides, radiographic studies also showed decreased density and porotic bone over both limbs and the body. Severe malnutrition impairs the tissue repair response to the disequilibrium state of the patient. Our patient presented toward a catabolic state with the development of body wasting. Increased nutritional demand with decreased nutritional intake was found in our patient. Severe malnutrition was also associated with impaired immune system, resulting in the patient’s being susceptible to infection in the pulmonary, genitourinary, and gastrointestinal tracts. Tissue changes in the patient resulted from thicker reduplicated basement membrane with enlarged endothelial cells. These further led to occlusion of capillaries and small vessels and disruption of skin microvasculature [69]. Skin functions as a barrier to protect against pathogens and mechanical stress, and temperature, and for peripheral circulation regulation, as well as a sensing organ, being impaired here. Pressure ulcers may increase the risk of contracture formation. However, pressure ulcers in our patient emerged due to the contracture in the bedridden condition. This pressure ulcer itself may aggravate the existing contracture through a pain and infection mechanism. In our case, we decided to perform a nonsurgical approach to the patient due to his general condition with these associated comorbidities.

## Conclusion

Prolonged immobility remains the most common cause of joint contracture. Besides, this condition is a common musculoskeletal finding following congenital conditions, chronic diseases, and traumatic accident. Physicians are faced with challenges in management due to intraoperative difficulties and problems, intraoperative and postoperative complications, inferior outcomes and results, and the need for an additional surgical procedure after the initial surgery. Chronic complications of patients with associated comorbidities may impair their prognosis and aggravate rehabilitative care, whether the patient undergoes a surgical procedure or not. Furthermore, psychological and socioeconomic conditions may become significant factors in regard to the recovery and rehabilitation of musculoskeletal disorders. These aspects include health beliefs, patient expectations and attitude to healing, educational level, and socioeconomic support. Furthermore, inadequate treatment at the first contact health center, late presentation, and missed injury following difficult access to healthcare facilities, adequate infrastructure, and trained manpower become the main problems related to the quality of healthcare, which may interfere with and aggravate the patient’s health condition. In addition, no fixed protocol or treatment has been established to date. The combination of surgery and rehabilitative care plays an important role in successful management of such cases. An evaluation of residual muscle strength, changes of bone density and characteristic, and the patient’s general and comorbid conditions must always be taken into account when considering the treatment choices. Eventually, a holistic approach was important in the management of this patient, in which not only physical, but also emotional, mental, spiritual, and social concerns associated with the patient must be comprehensively assessed.

## Data Availability

The data used to support the findings of this study are available from the corresponding author upon request.
